# The immunoregulatory role of integrins in pulmonary diseases

**DOI:** 10.3389/fimmu.2025.1716118

**Published:** 2025-12-10

**Authors:** Qing Xu, Chao Yang, Wanwen Li, Weiwei Qian, Shiyin Chen, Liqing Yang

**Affiliations:** 1 Department Respiratory and Critical Care Medicine, Sichuan Provincial People’s Hospital, School of Medicine, University of Electronic Science and Technology of China, Chengdu, China; 2 Medicine Surgery, Sichuan Provincial People’s Hospital, School of Medicine, University of Electronic Science and Technology of China, Chengdu, China; 3 Department of Thoracic Surgery, Sichuan Provincial People’s Hospital, School of Medicine, University of Electronic Science and Technology of China, Chengdu, China; 4 Emergency Department, Chengdu Shangjin Nanfu Hospital, Chengdu, Sichuan, China; 5 Department of Chinese Medicine, Sichuan Provincial People’s Hospital, School of Medicine, University of Electronic Science and Technology of China, Chengdu, China

**Keywords:** integrins, pulmonary diseases, immune cells, TGF-β pathway, extra cellular matrix, lung fibrosis, therapeutic targets, precision drug delivery

## Abstract

Integrins are a family of transmembrane adhesion receptors composed of α and β subunits that connect cells to the extracellular matrix and transmit biochemical and mechanical signals. They play a critical role in immune cell migration, maintenance of the alveolar-capillary barrier, and tissue repair. Pulmonary diseases often exhibit pathological features of immune imbalance, barrier disruption, and abnormal remodeling. Integrins, situated at the intersection of “cell-matrix-mechanical” signaling, exert decisive influence on disease progression by regulating mechanisms such as neutrophil and monocyte transendothelial migration, TGF-β activation, and the immune microenvironment. This review comprehensively summarizes the structural basis and bidirectional signaling mechanisms of integrins, along with their regulatory roles in the functions of pulmonary immune cells such as T cells, macrophages, and neutrophils. It emphasizes the pathological mechanisms of integrins in diseases including ARDS, pulmonary fibrosis, COPD, asthma, and lung cancer (particularly the dual role of the integrin-TGF-β axis in inflammation and fibrosis) It introduces current and emerging targeted therapeutic strategies, including α_v_β_6_ monoclonal antibodies, small-molecule antagonists, inhaled delivery, and biomimetic delivery approaches. We emphasize that balancing the suppression of pathogenic signals with the maintenance of tissue homeostasis is essential when targeting integrins for therapeutic intervention. Future progress will depend on developing more precise delivery technologies and patient stratification strategies to advance the translational application of integrin-targeted therapies across multiple pulmonary diseases.

## 1 Introduction

Integrins are a family of transmembrane receptors composed of heterodimeric α and β subunits. In mammals, there are 24 functional receptors formed by non-covalent pairing of 18 distinct α subunits with 8 distinct β subunits (1–3). Different αβ combinations determine specific ligand recognition and signal output: for example, theα_v_ family (α_v_β_1_, α_v_β_3_, α_v_β_5_, α_v_β_6_, α_v_β_8_, α_8_β_1,_ α_5_β_1_, and α_IIb_β_3_) (2, 3), the leukocyte-specific β_2_ family (α_L_β_2_, α_M_β_2_, α_X_β_2_, α_D_β_2_) (2), and the collagen/laminin-binding β_1_ family (α_1_β_1_, α_2_β_1_, α_6_β_1_, etc.) (2). Structurally, the extracellular domain of integrins contains a metal ion-dependent ligand-binding site (MIDAS) and head/leg domains (4, 5); their activity is achieved through controlled conformational transitions from “bent-closed” to “extended-closed” to “extended-open”. Intracellular “inside-out” activation depends on talon and kindlin binding to the β-tail to enhance affinity and clustering; “Outside-in” signaling activates pathways including Fyn, FAK, PI3K, and Rho GTPases following ligand binding and receptor clustering, coordinating with the actin cytoskeleton to mediate mechanical transduction (4, 6).

Under physiological conditions, integrins perform three core functions: (1) Adhesion and Tethering: Connecting the cytoskeleton to the extracellular matrix (ECM), maintaining the integrity of the alveolar-capillary barrier and airway epithelium (6–8); (2) Directional Migration and Tissue Localization: Mediating immune cell rolling, firm adhesion, transendothelial migration, and intragranular crawling to achieve immune surveillance and mucosal tissue residency (9–11); (3) Signal Integration and Tissue Repair: Perceives changes in ECM components and stiffness, coupling inflammatory, regenerative, and fibrotic programs (12–15). Additionally, certain integrins function as co-receptors for complement/coagulation factors, apoptotic cell clearance, and specific pathogen invasion, expanding their immunoregulatory spectrum (16, 17).

Globally, lung diseases continue to impose a heavy health and economic burden (18). In the acute setting, acute respiratory distress syndrome (ARDS) and severe pneumonia remain major causes of ICU mortality and long-term disability (19, 20). Chronically, chronic obstructive pulmonary disease (COPD) and asthma affect hundreds of millions of people, leading to high recurrence rates and substantial healthcare resource utilization (21, 22); interstitial lung diseases such as idiopathic pulmonary fibrosis (IPF) are characterized by progressive gas exchange impairment and poor prognosis (23, 24); lung cancer has long ranked as the leading cause of cancer-related mortality (25). These diseases share common features of intertwined immune dysregulation, barrier disruption, and tissue remodeling. Integrins occupy a pivotal intersection at the “cell-matrix-mechanics-signaling” nexus, exerting decisive influence on disease trajectories: β_2_ integrins dominate neutrophil and monocyte adhesion and migration, contributing to increased vascular permeability and tissue injury in ARDS (26, 27); α_v_β_6_, α_v_β_8_, and α_v_β_1_ drive epithelial-mesenchymal communication and fibroblast activation by activating latent TGF-β, serving as key gatekeepers of fibrosis (28); α_4_β_1_, α_E_β_7_, and other integrins regulate the recruitment and retention of mucosal immune cells, linking airway inflammation and remodeling in asthma and COPD (29); α_v_β_3_/β_5_ integrins on macrophages play a role in clearing apoptotic cells and promoting inflammatory resolution (30); In the tumor microenvironment, integrins influence immune cell infiltration, angiogenesis, and tumor cell migration, contributing to immune evasion and metastasis (31).Disease-Specific and Microenvironmental Roles of Integrins in Lung Pathophysiology and Cancer in **Table 1**.

**Table 1:** Integrins and their functional roles across major pulmonary diseases and the tumor microenvironment.

Disease	Key integrins	Primary functions	Mechanisms	Reference
ARDS	β_2_ integrins	Neutrophil & monocyte adhesion and migration	Promote transendothelial migration → increase vascular permeability → tissue injury	(26, 27)
Pulmonary Fibrosis	α_v_β_6_, α_v_β_8_, α_v_β_1_	Activate latent TGF-β; epithelial–mesenchymal crosstalk	Drive fibroblast activation and extracellular matrix deposition; key gatekeepers of fibrosis	(28)
Asthma/COPD	α_4_β_1_, α_E_β_7_ (and others)	Regulate immune cell recruitment & retention in mucosal tissues	Link airway inflammation to structural remodeling	(29)
Resolution of Inflammation	α_v_β_3_, α_v_β_5_ (on macrophages)	Promote efferocytosis and inflammatory resolution	Enhance clearance of apoptotic cells and restoration of tissue homeostasis	(30)
Tumor Microenvironment (TME)	Multiple integrins (e.g., α_v_β_3_, β_5_, β_1_ families)	Regulate immune infiltration, angiogenesis, tumor cell migration	Facilitate immune evasion, promote angiogenesis, and support metastasis	(31)

Due to the rapid advancement of single-cell omics, spatial transcriptomics, and *in vivo* imaging technologies, the cell lineage-specific expression, spatiotemporal dynamics, and force sensing of integrins within the lung tissue microenvironment are being progressively decoded. Concurrently, pharmacological intervention strategies targeting integrins are evolving from “pathway antagonism” toward “cell/organ-targeted delivery” and “temporally precise regulation”—including α_v_β_6_/α_v_β_1_ selective inhibitors (32), *de novo* designed high-affinity microproteins (33), inhalation-delivered small molecules/cyclic peptides (34), and layered therapeutic strategies coupled with fibrosis or immune markers (35). Simultaneously, balancing “inhibition of pathogenic signals” with “preservation of physiological repair,” and avoiding potential risks such as systemic immunosuppression and delayed bleeding/wound healing, remain critical scientific challenges for targeted integrin therapies.

Against this backdrop, this paper extensively summarizes the structural basis and activation mechanisms of integrins, along with their role in regulating the functions of pulmonary immune cells. It focuses on elucidating how integrins influence the migration, adhesion, phagocytosis, and activation of immune cells such as T lymphocytes, macrophages, and neutrophils. Subsequently, the paper will discuss integrin-mediated pathological mechanisms categorized by disease (ARDS, pulmonary fibrosis, COPD, asthma, lung cancer, etc.), with particular attention to the dual role of the integrin-transforming growth factor β (TGF-β) axis in inflammation and fibrosis. Finally, this paper will introduce current and emerging therapeutic strategies targeting integrins, including monoclonal antibodies, small-molecule antagonists, and novel drug delivery systems, while proposing future research directions and translational applications.

## 2 Structural basis and signaling mechanism of integrins

**Figure 1:**
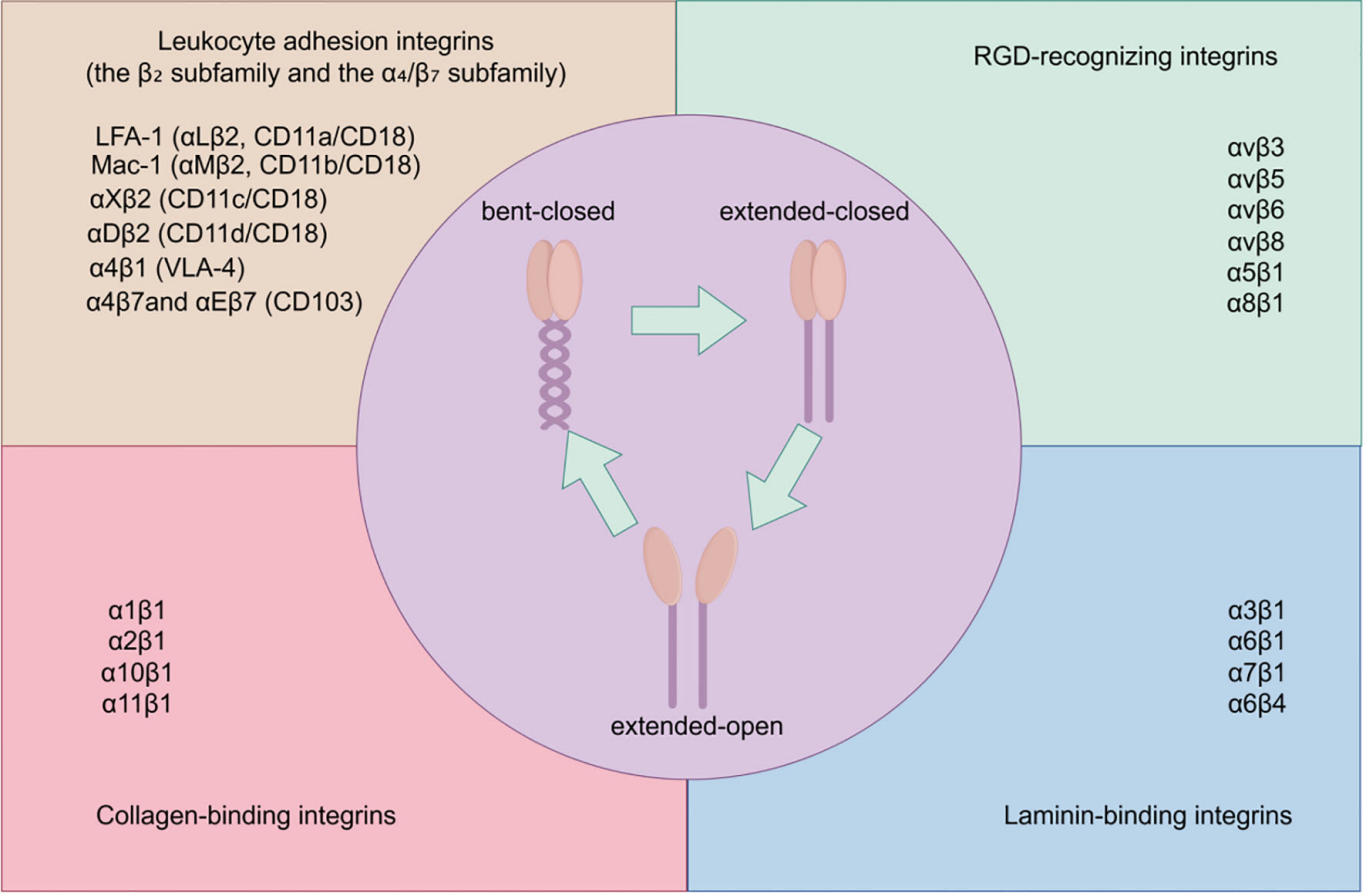
Classification and conformational states of integrins.

The structure of integrins determines their function. As heterodimeric receptors, the diverse subunit combinations within the integrin family confer distinct ligand specificity and downstream signaling capabilities. Simultaneously, the unique conformational switching mechanism of integrins enables them to act as a “switch” between intracellular and extracellular signals. This section will outline the classification and structural features of integrins, and introduce their conformational activation and bidirectional signaling mechanisms. The classification of integrins and their conformational transitions are presented in **Figure 1**.

Integrins are categorized into four major classes based on their ligand-binding specificity: leukocyte adhesion integrins (β_2_ subfamily and α_4_;/β_7_ subfamily), RGD-recognizing integrins, collagen-binding integrins, and laminin-binding integrins. Each quadrant lists representative integrins for their respective categories. The central schematic illustrates three typical conformational states of integrins: bent-closed, extended-closed, and extended-open, representing different levels of ligand affinity.

### 2.1 Family classification

Based on ligand-binding characteristics, integrins can be classified into four functional subfamilies: (1) Leukocyte adhesion integrins (β_2_ subfamily and α_4/β_7 subfamily), including LFA-1 (α_L_β_2_, CD11a/CD18), Mac-1 (α_M_β_2_, CD11b/CD18), α_X_β_2_ (CD11c/CD18), α_D_β_2_ (CD11d/CD18), and others such as α_4_β_1_ (VLA-4), α_4_β_7_, α_E_β_7_ (CD103). These primarily mediate adhesion between leukocytes and endothelial cells as well as cell-cell adhesion, playing roles in immune cell chemotaxis, adhesion, and migration (2, 36–38). (2) RGD-binding integrins, including typical members such asα_v_β_3_, α_v_β_5_, α_v_β_6_, α_v_β_8_, α_5_β_1_, α_8_β_1_, recognize the Arg-Gly-Asp (RGD) motif in ligands like fibronectin, vitronectin, and osteopontin (39). (3) Collagen-binding integrins (α_1_β_1_, α_2_β_1_, α_10_β_1_, α_11_β_1_) recognize the GFOGER motif on collagen, mediating interactions between the basement membrane and the stroma (40). (4) Laminin-binding integrins (α_3_β_1_, α_6_β_1_, α_7_β_1_, α_6_β_4_ etc.) mediate adhesion between epithelial cells and the basement membrane (41). Notably, in addition to classical ECM ligands, integrins can bind to cell surface receptors such as intercellular adhesion molecule-1 (ICAM-1) and vascular cell adhesion molecule-1 (VCAM-1), and serve as receptors for certain viral and bacterial toxins. This multi-ligand recognition property positions integrins as “molecular bridges” connecting immune cells to their surrounding microenvironment (42).

### 2.2 Structural conformation

Each integrin comprises an α subunit and a β subunit, both featuring large extracellular domains, a single transmembrane segment, and a short cytoplasmic tail (1–4). Approximately half of the α subunits contain an additional α_I_ (insertion) domain that directly participates in ligand binding (43, 44); while integrins lacking the α_I_ domain bind ligands through a binding pocket formed by the α-subunit’s β-helical paddle and the β_I_ domain (43). The affinity and functional state of integrins on the membrane are determined by their conformation: in the resting state, integrins adopt a bent-closed conformation, with the extracellular head folded and low ligand affinity (i.e., the “closed” state). Upon activation by endogenous signals (e.g., binding of cytoplasmic proteins talin or kindlin to the β-tail), integrins undergo conformational changes: first extending into an extended-closed conformation, then stabilizing into an extended-open conformation upon ligand binding, exhibiting high-affinity binding capacity (2, 45). An intermediate state termed bent-open is considered an activation intermediate for an alternative pathway (46). Overall, integrin conformational changes represent an inward-to-outward signal-induced process consolidated by ligand binding. The high-affinity open conformation often correlates with integrin clustering on the membrane surface, thereby enhancing the formation of structures such as focal adhesions and immune synapses.

### 2.3 Signal transduction

**Figure 2:**
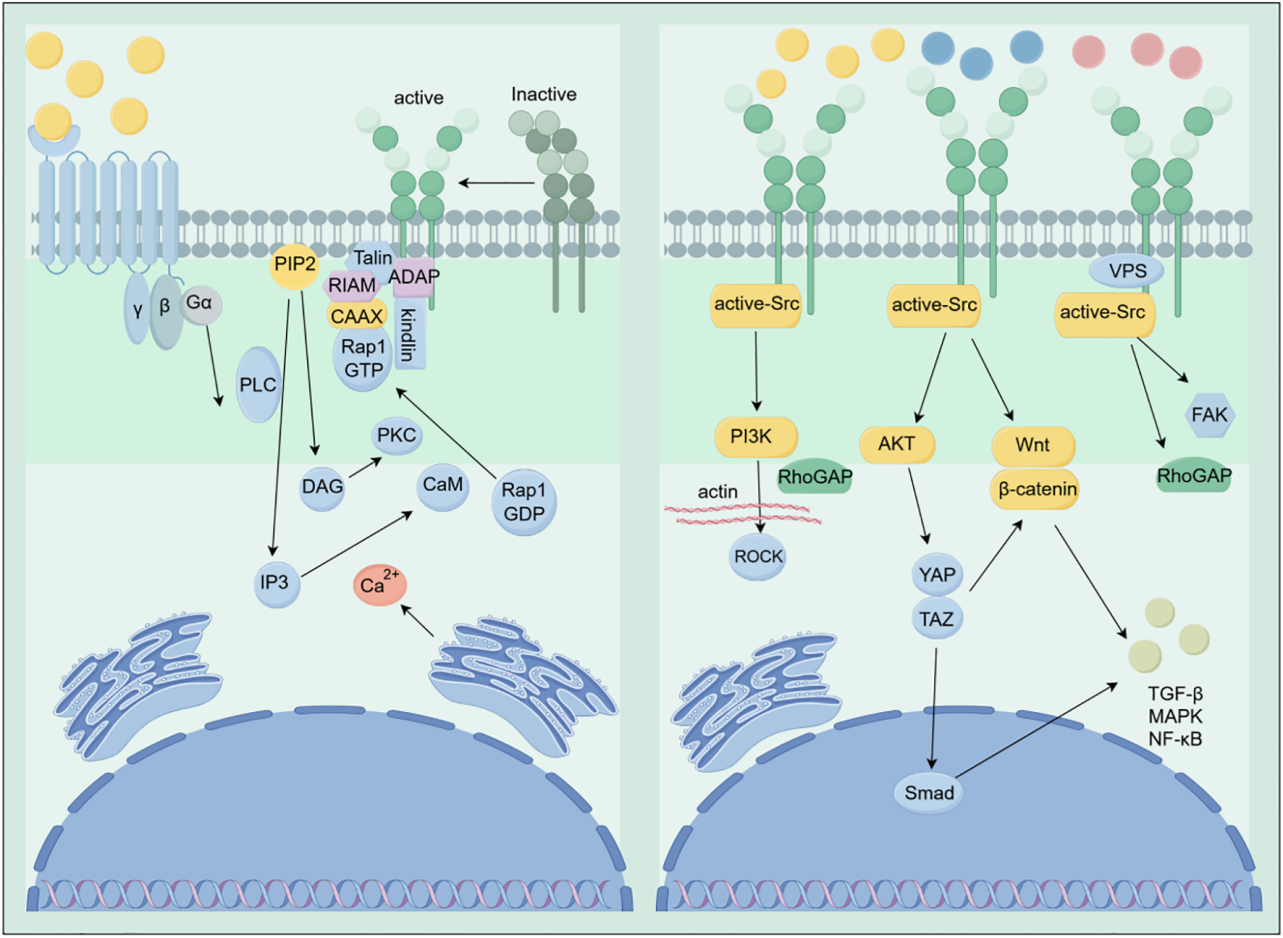
Schematic diagram of integrin activation and downstream signaling pathways. The left panel illustrates the inside-out activation process of integrin from an inactive to an active state: External signals activate PLC via G protein-coupled receptors (GPCRs), triggering the IP3/DAG pathway and Ca²^+^ release. PKC and CaM promote integrin activation mediated by the Talin/RIAM complex, while ADAP and Kindlin assist integrin activation. The right panel depicts the outside-in signal transduction following integrin activation: Activated integrins activate downstream signaling through Src family kinases, including PI3K, AKT, and FAK, which further regulate pathways involving RhoGAP, ROCK, β-catenin, YAP/TAZ, and Smad. This ultimately influences cytoskeletal reorganization, gene transcription, and TGF- β/MAPK/NF-κB-related cellular behaviors.

Integrins are cell adhesion receptors capable of bidirectional signal transduction across the cell membrane. This includes the “inside-out” mechanism, wherein intracellular signals activate integrins, and the “outside-in” mechanism, wherein integrin binding to the extracellular matrix triggers intracellular signaling cascades (47, 48). In inside-out activation, upstream signals from non-integrin receptors (e.g., GPCRs, tyrosine kinase receptors) promote binding of cytoplasmic proteins (e.g., talin, kindlin) to the integrin intracellular tail. This induces a conformational shift from a low-affinity bent state to a high-affinity extended state, enhancing integrin binding to ECM ligands (47). Conversely, in outward-signaling pathways, integrin clusters upon binding to extracellular matrix components (e.g., fibronectin, laminin, collagen). Conformation changes in the transmembrane domain and cluster formation recruit a series of focal adhesion-associated proteins and kinases to the intracellular tail, triggering multiple downstream signaling pathways. These bidirectional signaling pathways synergistically regulate cell adhesion, migration, and cellular responses to environmental cues. The integrin activation process and its downstream signaling networks are illustrated schematically in **Figure 2**.

#### 2.3.1 Classic signaling pathways following integrin activation and ECM binding

Upon binding to ECM ligands and clustering to form focal adhesions, the intracellular tail of integrins recruits key signaling molecules such as focal adhesion kinase (FAK) and Src non-receptor tyrosine kinases. The activated FAK/Src complex acts as a signaling hub, initiating multiple classical downstream pathways. These include the Ras-MAPK/ERK pathway (promoting cell proliferation and differentiation), the PI3K/AKT pathway (promoting cell survival, growth, and metabolism), and Rho family pathways mediated by GTPases (regulating actin reorganization and cell morphology) (49).Concurrently, the adaptor protein functions of FAK and Src recruit adhesion signaling mediators like Shc to the nucleus, inducing gene expression changes. For instance, activated FAK activates Ras via the Grb2/SOS complex, triggering a MAPK cascade that activates the transcription factor AP-1 to regulate gene expression. FAK/Src can also activate PI3K, leading to AKT phosphorylation, thereby influencing the activity of transcription factors like FOXO and inhibiting apoptosis. The RhoA/ROCK pathway is activated by integrins via FAK or integrin-linked kinase (ILK), promoting stress fiber formation and enhancing cellular contractile force (50, 51). Notably, integrin downstream signaling also encompasses inflammatory pathways like NF-κB: integrin-mediated adhesion activates the IκB kinase complex (IKK), inducing NF-κB nuclear translocation and triggering pro-inflammatory gene expression (52, 53). For instance, in rheumatoid arthritis models, activation of β_2_ and β_7_ integrins not only mediates immune cell migration to inflammatory sites but also exacerbates joint inflammation and destruction by triggering NF-κB and JNK pathways to promote proinflammatory cytokine release (54, 55). In summary, the classical signaling network triggered by integrin-ECM interactions involves the focal adhesion signaling complex centered on FAK/Src, which connects and activates pathways including PI3K/AKT, MAPK, RhoA/ROCK, and NF-κB. This network consequently influences diverse cellular processes such as survival, proliferation, division, migration, and inflammatory responses.

#### 2.3.2 Mechanical force sensing by integrins and YAP/TAZ activation

As one of the primary mechanical sensors in cells, integrins convert mechanical signals from the extracellular matrix into intracellular biochemical signals. Integrins link the ECM to the cytoskeleton. When cells experience stretching, compressive stress, or increased culture substrate stiffness, integrin clustering intensifies connections with actin filaments, transmitting external mechanical forces into the cell. Signaling pathways activated by integrin clustering—such as FAK/Src—further promote RhoA activation. RhoA, through its effector protein ROCK, facilitates stress fiber formation and generates contractile tension. Enhanced cellular tension inhibits the Hippo pathway via LATS1/2, leading to the dephosphorylation and release of downstream YAP/TAZ. Dephosphorylated YAP/TAZ readily translocates into the nucleus, where it binds transcription factors like TEAD to initiate the transcriptional programs of numerous mechanosensitive genes (56, 57). This mechanotransduction axis enables integrins to sense matrix stiffness: on soft substrates, insufficient tension keeps YAP/TAZ inactivated in the cytoplasm, whereas on stiffer substrates, YAP/TAZ more readily translocates to the nucleus to exert its effects. The integrin-cytoskeleton-nuclear continuum also directly transmits mechanical signals to the nucleus, inducing changes in chromatin conformation and gene expression. Experimental evidence indicates that both cell morphology and matrix mechanics influence the activation of transcription factors like YAP/TAZ and NF-κB: for example, increased matrix stiffness leads to greater nuclear translocation of YAP in fibroblasts, upregulating fibrotic genes like α-SMA and type I collagen (58). Concurrently, it enhances NF-κB nuclear localization via the Rho pathway, promoting expression of inflammatory factors such as IL-1β and IL-8 (59). Furthermore, integrin-mediated mechanical stimuli can activate non-canonical pathways like the JNK pathway via Src-FAK. JNK-activated c-Jun/AP-1 also participates in regulating mechanoresponsive genes (60, 61). In summary, integrins function as mechanical sensors, perceiving external forces and matrix stiffness through the focal adhesion complex. They exert mechanosensitive control over terminal effects including cell proliferation, survival, differentiation, fibrosis, and inflammation via RhoA-ROCK-mediated tension regulation pathways (62, 63).

#### 2.3.3 Cross-regulation of integrin signaling with TGF-β, Wnt, and other signals

Integrin signaling exhibits extensive cross-integration with multiple developmental and growth factor pathways, jointly regulating cellular fate decisions. For instance, in the TGF-β pathway, integrins play a crucial role in activating TGF-β (64). TGF-β is typically stored in the ECM as latent complexes (LAP/LTBP-coated forms), requiring release of the mature ligand to activate its receptor/SMAD signaling (65). Numerous integrins (e.g., α_v_β_1_, α_v_β_3_, α_v_β_5_, α_v_β_6_, α_v_β_8_) bind to LAP, with α_v_β_6_ and α_v_β_8_ being particularly important for mediating latent TGF-β activation (33). For instance, post-injury epithelial cells highly express α_v_β_6_ integrin, which binds and tugs on LAP within the ECM, triggering TGF-β release and activation. This induces epithelial-mesenchymal transition (EMT) and tissue fibrosis (66, 67). Conversely, TGF-β signaling also feedback-regulates the expression levels of integrins and other adhesion molecules, thereby influencing cellular adhesion and migration capabilities (12). In cancer, TGF-β and integrins synergistically promote invasion and metastasis: for instance, in triple-negative breast cancer, tumor cells activate TGF-β signaling via α_v_β_6_ integrin, inducing upregulation of the transcription factor SOX4 to facilitate immune evasion (68); In colorectal cancer, α_v_β_6_ on tumor cells activates paracellular fibroblasts via TGF-β, which then secrete SDF-1 to stimulate cancer cell migration and invasion (69). Thus, the integrin-ECM axis and TGF-β/SMAD pathway form a positive feedback loop, jointly driving processes such as EMT, cell migration, and tissue remodeling.

The interaction between the integrin and Wnt/β-catenin pathways also plays a crucial role in cellular development and tumorigenesis. Focal adhesion kinase (FAK) not only serves as a downstream signal of integrins but also acts as a co-regulator of the Wnt pathway: studies indicate that FAK activity influences the fate of epidermal stem cells through crosstalk with Wnt/β-catenin signaling. Regarding tumor invasion, integrin signaling can synergize with the Wnt pathway to induce EMT. For instance, the transcription factor Twist promotes EMT and motility in cancer cells through a complex network involving the β_1_ integrin–FAK/ILK axis and PI3K/AKT, MAPK/ERK, and Wnt signaling (70–72). Among these, β_1_ integrin, as the primary fibronectin/collagen receptor, serves as a key activator of FAK and ILK pathways. It enhances Wnt/β-catenin signaling through downstream cascades, thereby stabilizing EMT transcription programs like Snail and promoting tumor cell migration. Additionally, integrin-activated FAK/Src can cross-activate growth factor receptors (e.g., EGFR) or their downstream effectors, amplifying the impact of Wnt and TGF-β signaling on cellular behavior (73). This tight coupling between adhesion and growth factor signaling ensures coordinated cellular responses to microenvironmental changes. In developmental contexts, wound healing, and carcinogenesis, the cross-regulation between integrins and pathways like TGF-β, Wnt, and Notch influences cellular adhesion, motility, proliferation, differentiation status, and even stem cell fate, playing a crucial role in both tissue homeostasis and dysregulation (74).

## 3 Mechanisms of integrin action in different pulmonary diseases

Integrins play a pivotal role in the pathogenesis and progression of pulmonary diseases. Their effects extend beyond individual cells or single pathways, profoundly influencing the entire disease spectrum through multidimensional immune regulation, alterations in barrier function, and signaling networks. In acute respiratory distress syndrome (ARDS) and acute lung injury (ALI), integrin-mediated neutrophil adhesion and extravasation, monocyte recruitment, and increased endothelial permeability constitute key pathways amplifying acute inflammation (75). In interstitial lung diseases like idiopathic pulmonary fibrosis (IPF), integrins such as α_v_β_6_ and α_v_β_1_ activate latent TGF-β, driving fibroblast activation and collagen deposition—core drivers of fibrosis formation (76). In chronic airway inflammatory diseases like chronic obstructive pulmonary disease (COPD) and asthma, leukocyte integrins mediate immune cell infiltration, while α_v_β_6_/α_v_β_8_ promote airway remodeling through abnormal TGF-β activation, ultimately leading to airflow limitation and tissue structural remodeling (77, 78). Furthermore, in lung cancer, integrins not only regulate tumor cell adhesion, invasion, and metastasis but also reshape the tumor immune microenvironment, influencing immune cell infiltration and immunotherapy response (79). Notably, in infectious pneumonia, integrins participate in pathogen clearance by neutrophils while potentially exacerbating tissue damage through excessive activation, demonstrating a dual “protective-harmful” effect (80). In summary, integrins exhibit a “common mechanism + disease-specific pathway” pattern across diverse pulmonary pathologies. They collectively influence disease progression through adhesion/migration and TGF-β signaling regulation, while simultaneously generating distinct pathological phenotypes due to cell type and microenvironmental variations. This cross-disease consistency and heterogeneity provides a theoretical foundation for deepening our understanding of disease mechanisms and developing targeted therapeutic strategies. Representative integrin subunits and their key mechanisms involved in different pulmonary diseases are summarized in **Table 1**, providing an intuitive overview of the pivotal roles of integrins in inflammation, fibrosis, airway remodeling, and tumor progression. Integrin Subunit Networks and Mechanistic Signaling Pathways in Pulmonary Disease in **Table 2**.

**Table 2:** Integrin subunits and mechanistic pathways in lung disease.

Disease	Integrin subunits	Key mechanisms	Reference
ARDS/ALI	β_2_; LFA-1 (α_L_β_2_), Mac-1 (α_M_β_2_);	Mediate neutrophil firm adhesion and extravasation	(81, 82)
	α_4_β_1_;	Monocyte recruitment via α_4_β_1_–VCAM-1	(83)
	endothelial α_v_β_5_	Endothelial α_v_β_5_ increases permeability via actin stress fibers → edema	(84, 85)
Idiopathic pulmonary fibrosis (IPF)	Epithelial α_v_β_6_/α_v_β_8_;	α_v_β_6_/α_v_β_8_ recognize LAP (RGD) and activate latent TGF-β via cytoskeletal traction	(86)
	myofibroblast αvβ1;	α_v_β_1_ sustains TGF-β activation and ECM deposition	(87)
	immune α_M_β_2_	α_M_β_2_ participates in mechanosensing and pro-fibrotic polarization ECM stiffness forms a mechano–TGF-β positive feedback loop driving collagen accumulation	(14, 88)
Asthma	Eosinophil/Th2: α_4_β_1_;	Eosinophils and Th2 lymphocytes migrate into the airway via α_4_β_1_–VCAM-1 binding	(89, 90)
	β_2_ integrin (Mac-1, CD11b/CD18)	Eosinophils adhere to periostin via Mac-1, promoting their retention in airway tissues	(91)
	α_v_β_6_	LPA_2_ receptor signaling induces α_v_β_6_-mediated TGF-β activation	(92)
COPD	Epithelial α_v_β_6_/α_v_β_8_; myofibroblast	Excess α_v_β_6_/α_v_β_8_ activation of TGF-β drives small-airway fibrosis/EMT	(93, 94)
	β_2_; macrophage α_D_β_2_	β_2_ promotes neutrophil extravasation and protease release	(95)
Lung cancer (NSCLC/SCLC)	Tumor α_v_β_6_/α_v_β_3_/α_v_β_5_,	Activates TGF-β to promote EMT/invasion	(96, 97)
	α_v_	Inhibits the infiltration and function of cytotoxic T cells into tumors	(98)

### 3.1 ARDS and acute lung injury

ARDS is an acute diffuse lung injury caused by factors such as infection and trauma, remaining a major cause of ICU mortality and long-term disability worldwide. Its pathological features include increased pulmonary capillary permeability, pulmonary edema, and severe inflammatory cell infiltration, with massive neutrophil accumulation considered one of the primary causes of alveolar damage. Integrins participate in multiple stages of ARDS pathogenesis, significantly influencing disease progression:

In the early phase of ARDS, activated neutrophils rapidly adhere to pulmonary capillary endothelium via β_2_ integrin and transmigrate into the alveolar space (99). Excessive neutrophil recruitment releases proteases and reactive oxygen species, disrupting endothelial and epithelial barriers. This leads to alveolar space filling with edematous fluid, exacerbating hypoxemia (100). In animal models, administration of anti-CD18 antibodies or ICAM-1 blockade significantly reduced neutrophil extravasation and pulmonary edema, indicating that the β_2_ integrin-ICAM pathway is a primary route for neutrophil infiltration and tissue injury in ARDS (81, 82). Notably, enzymes such as elastase released during neutrophil-endothelial adhesion can also disrupt intercellular junctions via Mac-1-mediated “bridging,” further amplifying increased permeability and inflammatory responses (101). Thus, inhibiting neutrophil integrin adhesion holds promise for mitigating acute injury in ARDS.

In addition to neutrophils, monocytes and macrophages also accumulate extensively in the lungs during ARDS. Research indicates that monocyte-macrophages primarily enter the pulmonary interstitium through VLA-4 integrin binding to VCAM-1. In sepsis-associated ARDS, extracellular vesicles (EC-EVs) released by damaged endothelial cells carry high levels of VCAM-1 on their surface. These EC-EVs can target monocytes and reprogram them into pro-inflammatory macrophages, exacerbating lung injury. Blocking α_4_ integrin on monocytes or clearing these vesicles attenuates this effect (83). This reveals a novel mechanism: injured endothelium remotely drives secondary injury mediated by monocyte-macrophages via integrin ligand (VCAM-1) signaling. Concurrently, during the recovery phase of ARDS, macrophages exert protective effects through integrins—for example, utilizing MerTK and α_v_β_3_/β_5_ to recognize and phagocytose apoptotic neutrophils, thereby promoting inflammation resolution and tissue repair (102). Thus, integrins participate in both the vicious cycle of inflammation amplification and the positive feedback of inflammation resolution in ARDS.

Pulmonary vascular leakage in acute lung injury directly causes fatal pulmonary edema. Recent studies reveal that α_v_β_5_ integrin on pulmonary endothelial cells is a core regulator of increased vascular permeability (85). In ischemia-reperfusion and ventilator injury models, functional blockade of α_v_β_5_ significantly suppressed pulmonary capillary leakage (103). α_v_β_5_−/− mice exhibited no significant vascular leakage under high mechanical stretch ventilation. Knocking out or blocking endothelial α_v_β_5_ attenuated VEGF, TGF-β, and thrombin-induced increases in endothelial permeability and inhibited stress fiber formation. This indicates that α_v_β_5_ promotes endothelial cell contraction and gap opening by interacting with the cytoskeleton (84, 85). In summary, the α_v_β_5_ integrin is a key contributor to endothelial barrier dysfunction in ARDS, and targeting it offers a novel approach for preventing and treating pulmonary edema.

### 3.2 Pulmonary fibrosis (e.g., idiopathic pulmonary fibrosis)

Pulmonary fibrosis is characterized by progressive scarring of the pulmonary interstitium. For instance, idiopathic pulmonary fibrosis (IPF) is a fatal fibrotic disease with unknown etiology and extremely poor prognosis. One core driver of fibrosis is the sustained activation of TGF-β signaling, which induces fibroblast proliferation, differentiation, and excessive production of ECM components such as collagen. The integrin-TGF-β axis plays a pivotal role in this process (104). Under normal conditions, TGF-β exists in tissues as an inactive latent complex, requiring release of active TGF-β to exert its effects (28, 105). Integrins of the α_v_ family (particularly α_v_β_6_ and α_v_β_8_) bind to the RGD sequence on latent TGF-β complexes, inducing their activation and thereby initiating the fibrosis cascade. In pulmonary fibrosis, abnormal expression and activation of integrins lead to uncontrolled TGF-β signaling, accompanied by alterations in the immune microenvironment (86).

Alveolar epithelial cells highly express integrin α_v_β_6_, currently recognized as one of the most critical TGF-β activation factors. While α_v_β_6_ expression is low in healthy tissues, it is significantly upregulated in the alveolar epithelium of IPF patients, with its expression levels closely correlated with disease progression and poor prognosis (106). Mouse models and clinical trials demonstrate that genetic knockout of α_v_β_6_ or administration of selective α_v_β_6_ monoclonal antibodies (e.g., BG00011) can prevent bleomycin-induced pulmonary fibrosis and even reverse established fibrosis (76, 107). This conclusively demonstrates the pivotal role of α_v_β_6_-mediated TGF-β activation in the pathogenesis of pulmonary fibrosis. Mechanistically, α_v_β_6_ integrins localize to the surface of damaged epithelial cells, where they bind latent TGF-β and, through cytoskeletal traction, cleave LAP to release active TGF-β1 (108, 109). The latter promotes the transformation of adjacent fibroblasts into myofibroblasts and the deposition of ECM, forming fibrous foci (110). However, it is important to emphasize the “double-edged sword” effect of the integrin-TGF-β axis: moderate α_v_β_6_-TGF-β signaling helps limit inflammation and maintain epithelial integrity, while complete inhibition of this pathway may release the brake on inflammation. For example, α_v_β_6_ knockout mice exhibit hyperactivated alveolar macrophages, increased elastase activity, and spontaneous emphysema-like changes. This suggests that anti-fibrotic therapies must balance the suppression of fibrosis with the maintenance of fundamental immune homeostasis (111).

Fibroblasts and myofibroblasts also express multiple integrins, participating in the maintenance of the fibrotic microenvironment. Of particular note is integrin α_v_β_1_, which is primarily distributed on myofibroblasts and is considered an alternative TGF-β activation pathway. Studies reveal that α_v_β_1_ is similarly upregulated in the lungs of IPF patients, with its high expression correlated to the formation of fibrotic foci (87). Small-molecule inhibitors (such as pan-α_v_ antagonists) blocking α_v_β_1_ can reverse established fibrosis in mouse models, though caution is warranted in interpretation due to selectivity issues. β_1_ integrins lacking α_v also participate in fibrosis. Preclinical evidence indicates roles for α_3_β_1_, α_4_β_1_ and α_8_β_1_ in pulmonary fibrosis (76). However, due to the lack of efficient tools targeting these integrins, no related clinical development is currently underway (112). Additionally, α_v_β_8_ integrin has been reported to play a significant role in small airway fibrosis associated with COPD and asthma (77). Overall, different integrins and cell types contribute differently across distinct phases of fibrosis: α_v_β_6_ on epithelial cells primarily triggers fibrosis initiation in the early stage, while α_v_β_1_ on myofibroblasts sustains the expansion of fibrotic foci in the later stage.

Fibrotic lung tissue contains a large number of infiltrating macrophages and lymphocytes, whose interactions with fibroblasts also influence disease progression. Regarding macrophages, recent *in vitro* co-culture studies demonstrate that when pro-fibrotic macrophages are co-cultured with fibroblasts on a rigid substrate, macrophages sense the stiff mechanical environment via integrins and become activated. This tripartite arrangement reproduces extensive fibrotic ECM deposition (113). The antifibrotic drug pirfenidone interferes with this mechanosensitive activation of macrophages by inhibiting their α_M_β_2_ integrin (Mac-1) and ROCK2 signaling, thereby reducing fibrosis progression (14, 88). This indicates that, beyond epithelial-mesenchymal signaling, immune cells mediate fibrosis through integrin-mediated mechanical and inflammatory signaling, representing a potential regulatory axis. Regarding lymphocytes, TGF-β in chronic inflammation exerts immunosuppressive effects—integrin activation of TGF-β not only drives fibrosis but also suppresses anti-fibrotic immune responses such as Th1/CTL activity (12, 114). Consequently, fibrotic tissues often exhibit an immunosuppressive microenvironment, making fibrosis more difficult to reverse.

### 3.3 Asthma

Asthma is a heterogeneous disease characterized by reversible airway obstruction, chronic airway inflammation, and airway hyperresponsiveness (AHR), affecting hundreds of millions of people worldwide (115). Pathological changes include eosinophil-predominant inflammatory infiltration, Th2-type immune responses, and remodeling such as airway smooth muscle hypertrophy and subepithelial fibrosis. Integrins play roles in multiple aspects of asthma pathogenesis:

Inflammatory cell recruitment: Eosinophils and Th2 lymphocytes are key effector cells in allergic asthma inflammation. Eosinophil migration from the bloodstream to the bronchial mucosa primarily depends on α_4_β_1_ integrin (VLA-4) binding to V-cell chemotactic antigen-1 (VCAM-1) on activated endothelial cells (90). In allergic asthma animal models, anti-α_4_β_1_ antibodies significantly reduce eosinophil recruitment to the lungs and attenuate airway inflammation (89). Concurrently, eosinophils themselves express β_2_ integrins (e.g., Mac-1) (116). Recent studies suggest that Mac-1 on eosinophil surfaces may bind to the airway matrix protein periostin, facilitating eosinophil retention within airway tissues (91). Periostin is a matrix molecule secreted by airway epithelium under Th2 inflammation. Thus, eosinophils achieve “settlement” at inflammatory sites through integrin-ligand interactions, subsequently releasing toxic granules and inflammatory mediators that cause airway epithelial damage and hyperresponsiveness (117). For lymphocytes such as Th2 cells, α_4_β_1_ integrin similarly mediates their directed migration into the lungs. Blocking this pathway reduces lymphocyte infiltration in the lungs of sensitized mice (89).

Airway Remodeling and Integrin Activation: Chronic inflammation not only causes airway narrowing but also induces irreversible structural remodeling by triggering integrin signaling in epithelial and stromal cells. In bronchial tissue from asthma patients, inflammatory mediators such as IL-1β synergize with integrin signaling to promote the expression of matrix genes including collagen (118). Additionally, lysophosphatidic acid (LPA) secreted by airway epithelium can induce TGF-β activation via α_v_β_6_ integrin through LPA_2_ receptors, further stimulating fibroblasts to produce active ECM (92). This creates a vicious cycle: inflammation promotes integrin upregulation, integrins activate TGF-β, TGF-β drives remodeling, and remodeling creates a favorable environment for inflammatory cell retention. This explains why anti-inflammatory treatments alone (e.g., corticosteroids) struggle to completely halt the remodeling process. Therefore, breaking this cycle requires a dual-pronged approach: simultaneously suppressing inflammation and intervening in the integrin-TGF-β pathway.

### 3.4 Chronic obstructive pulmonary disease

COPD encompasses pathological types such as emphysema and chronic bronchitis, characterized by progressive airflow limitation accompanied by irreversible parenchymal destruction and small airway remodeling. With complex etiology, COPD affects hundreds of millions globally and is associated with high recurrence rates and significant healthcare burden. Its development is closely linked to chronic inflammation and tissue remodeling, where integrins exert complex regulatory roles:

Emphysema manifests as destruction of alveolar septa and diminished elastic recoil, often associated with protease dysregulation caused by chronic inflammation. Under normal conditions, α_v_β_6_ integrin on alveolar epithelial cells maintains a “baseline anti-inflammatory” state through low-level activation of TGF-β, thereby inhibiting excessive protease release by macrophages and protecting alveolar structure. However, in COPD patients, chronic stimuli such as smoking may impair α_v_β_6_ function or downregulate its expression in epithelial cells. This weakens TGF-β signaling inhibition, leading to excessive activation of macrophages and neutrophils, increased destructive enzymes like elastase, and persistent erosion of alveolar structure (93, 94). This concept is supported by animal studies: α_v_β_6_-deficient mice spontaneously develop elastic fiber destruction and alveolar space enlargement, resembling emphysematous changes (119). Therefore, it can be inferred that maintaining moderate activity of the α_v_β_6_-TGF-β axis may help prevent excessive alveolar structural destruction in the prevention and treatment of emphysema. Naturally, precisely regulating this pathway within the inflammatory environment of COPD to simultaneously avoid fibrosis and inhibit emphysema progression represents a critical direction for future research.

Another pathological feature of COPD is the deposition of submucosal fibrosis and smooth muscle hyperplasia in small airways, leading to airway narrowing. This is associated with an imbalance in the epithelial-mesenchymal transition unit (EMTU) under chronic inflammatory stimulation (120). Studies indicate that small airway epithelial cells also express integrins α_v_β_8_ and α_v_β_6_ (7). When chronic stimuli (such as recurrent infections or smoking) abnormally enhance TGF-β activation mediated by these integrins, it induces excessive ECM production by small airway fibroblasts, leading to bronchial wall thickening and reduced compliance. Notably, α_v_β_8_ integrin has been found to be upregulated in COPD small airway fibrosis and drives local TGF-β release (121). Blocking α_v_β_8_ holds promise for reducing fibrotic deposition in small airways (122). Furthermore, macrophages in the lungs of chronic smokers may upregulate α_D_β_2_ integrin, enhancing their adhesion to inflammatory sites and aggregation. These macrophages can secrete pro-fibrotic factors like TGF-β to act on airway fibroblasts (95). Further studies indicate that pulmonary fibroblasts expressing integrin α_8_β_1_ also promote fibrotic gene expression under hypoxic microenvironments. α_v_β_8_-MMP-14-mediated protease-dependent TGF-β activation constitutes a key pathway in small airway fibrosis, complementing the traction-dependent α_v_β_6_ model (123). These findings indicate that multiple integrins collectively contribute to small airway remodeling within the chronic inflammatory state of COPD. Clinically, this remodeling exacerbates airway resistance, leading to irreversible respiratory function decline.

Throughout the course of COPD, immune cells such as neutrophils, macrophages, and lymphocytes undergo prolonged infiltration into the airways and pulmonary interstitium. Their recruitment and settlement mechanisms are intrinsically linked to integrins. For instance, neutrophils enter the lung tissue of COPD patients via LFA-1 and Mac-1, releasing collagenase and elastase during this process that degrade the matrix and exacerbate lung tissue destruction (124). Neutrophil-dominant inflammation further attracts additional neutrophils by releasing chemokines like IL-8, creating a vicious cycle. One role of integrins in this cycle is to delay neutrophil apoptosis: ICAM-1 binding to β_2_ integrin in the inflammatory microenvironment provides survival signals, prolonging neutrophil lifespan and sustaining pro-inflammatory effects (125). Regarding macrophages, those in COPD lungs are often polarized toward the M1 (pro-inflammatory) phenotype and secrete proteases causing tissue injury. Interaction between Mac-1 and damaged tissue components may be a factor sustaining this M1 phenotype. In later stages of COPD, as tissue hypoxia and destruction progress, some macrophages switch to the M2 phenotype to participate in clearing cellular debris and repair; at which point α_v_β_3_/β_5_ integrins mediate their phagocytosis of apoptotic cells, aiding in inflammation resolution and tissue stabilization (126, 127). Thus, integrins coordinate the recruitment and functional switching of immune cells in COPD, influencing both disease progression and remission.

### 3.5 Lung cancer

Lung cancer has long ranked as the leading cause of cancer-related deaths worldwide. During tumor progression, interactions between cancer cells and the tumor microenvironment (including immune cells and stromal cells) determine tumor growth, invasion, and immune evasion. Integrins play a pivotal role in tumor behavior due to their dual function in cell adhesion and signaling. Their effects on lung tumorigenesis, progression, and anti-tumor immune regulation primarily include the following aspects:

Tumor cells often acquire enhanced infiltration and metastatic capabilities by altering adhesion molecule expression. In non-small cell lung cancer (NSCLC), many tumor cells overexpress integrin α_v_β_6_. This integrin not only enhances tumor cell binding to matrix components like fibronectin, promoting invasion and spread, but also activates TGF-β to create an immunosuppressive microenvironment. Specifically, α_v_β_6_ mediated TGF-β induces increased regulatory T cells within tumors while suppressing effector T cell function, thereby aiding tumor escape from immune clearance (96). Additionally, α_v_β_3_ and α_v_β_5_ integrins play roles in tumor angiogenesis and metastasis. Their overexpression enhances tumor cell interactions with the basement membrane and vascular matrix, boosting cancer cells’ ability to detach from their original site and enter the circulation (97). Studies indicate that blocking α_v_β_3_ inhibits M2 macrophage-mediated invasion and migration of NSCLC cells. Simultaneously, adhesion between tumor-associated macrophages (TAMs) and tumor cells also depends on integrins. In lung adenocarcinoma models, M2 macrophages bind to β_2_ integrin on tumor cells via ICAM-1, facilitating cancer cell detachment from cell clusters and increasing metastatic potential (128). Thus, integrins not only confer invasive capacity to tumor cells themselves but also act as accomplices in tumor-macrophage “collusion.

The lung cancer microenvironment harbors a large number of immune cells, including T cells, NK cells, tumor-associated macrophages, and neutrophils. Integrins influence the distribution and state of these immune cells within tumors. Regarding lymphocytes, tumor-expressed integrin α_v_ modifies the phenotype of tumor-infiltrating lymphocytes (TILs) via the TGF-β pathway. Studies comparing lung cancer patients treated with immune checkpoint inhibitors revealed that those with low tumor cell α_v_ expression exhibited better prognosis and higher intratumoral density of CD8^+^CD103^+^ resident memory T cells (Trm) (98). Mechanistically, tumor cell α_v_ activates TGF-β, inducing CD8^+^ T cells to express integrin α_E_β_7_ (CD103) and adopt a resident phenotype. Although these Trm cells express granzyme B, they may be functionally constrained within a TGF-β-dominant suppressive environment (129). Therefore, inhibiting tumor cell α_v_ integrin (or its downstream TGF-β signaling) holds promise for enhancing anti-PD-1 therapy efficacy by enabling more cytotoxic T cells to infiltrate tumors and exert their effects. Second, regarding macrophages, integrins determine their polarization and function. Tumor cells overexpressing β_8_ integrin are expected to drive nearby macrophages toward M2 polarization by secreting the chemokine CCL5 (130). Conversely, M2 macrophages release IL-8 and other factors that promote tumor growth and angiogenesis (131). Blocking tumor β_8_ or its CCL5 signaling partially reverses macrophage polarization and inhibits tumor progression. Furthermore, integrins expressed by certain myeloid cells also contribute to immunosuppression. For instance, α_4_β_1_ integrin on myeloid-derived suppressor cells (MDSCs) binds tumor-derived molecules like IL-1β and SDF-1α, promoting MDSC accumulation within tumor tissues and suppressing T cell function (132, 133). Thus, within the lung cancer microenvironment, integrins constitute a critical interface for “dialogue” between tumor cells and immune cells, regulating the balance between immune attack and immune escape.

### 3.6 Pulmonary hypertension

Emerging evidence has established a critical role for integrin signaling in the development and progression of pulmonary hypertension (PH). Integrins modulate endothelial dysfunction, extracellular matrix (ECM) remodeling, and pulmonary artery smooth muscle cell (PASMC) proliferation—three core pathological processes underlying pulmonary vascular remodeling (134). In particular, integrin αvβ3 is markedly upregulated in remodeled pulmonary arteries and actively promotes PASMC migration and proliferation through FAK/Src–ERK signaling, contributing to medial thickening and increased vascular resistance (135). In parallel, integrin α5β1 regulates fibronectin-dependent mechanotransduction, ECM stiffness, and downstream pro-proliferative signaling, thereby facilitating persistent vascular remodeling and PH progression (136). These mechanistic insights highlight integrins as central regulators of PH pathology and support their potential as therapeutic targets in vascular remodeling.

## 4 Therapeutic strategies targeting integrins

**Figure 3:**
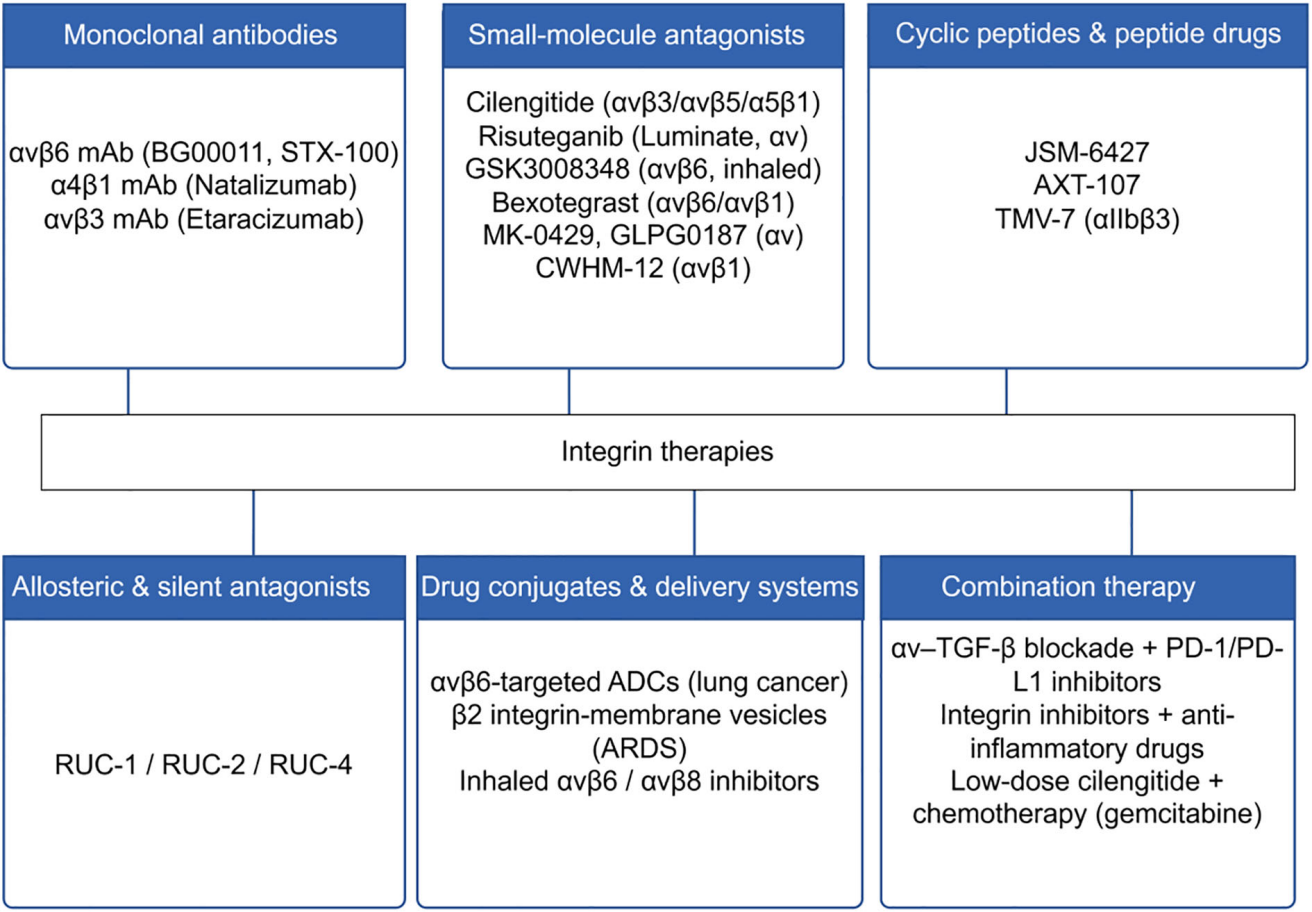
Therapeutic strategies targeting integrins.

Integrin-targeting drugs come in diverse forms, including monoclonal antibodies (such as α_v_β_6_ mAb BG00011, α_4_β_1_ mAb Natalizumab, α_v_β_3_ mAb Etaracizumab), small-molecule antagonists (such as Cilengitide, Risuteganib, GSK3008348, Bexotegrast), cyclic peptides and peptide drugs (e.g., JSM-6427, AXT-107, TMW-7). Additionally, these include allosteric and silencing antagonists (RUC-1/2/4), drug conjugates and delivery systems (e.g., α_v_β_6_-targeted ADCs, β_2_ integrin-targeting vesicular systems, inhaled α_v_β_6_/α_v_β_8_ inhibitors), and combination therapeutic strategies (e.g., αv–TGF-β blockade combined with PD-1/PD-L1 inhibitors, integrin inhibitors combined with anti-inflammatory drugs, low-dose Cilengitide combined with the chemotherapy drug gemcitabine).The various categories of integrin-targeted therapeutics, along with representative emerging agents, are schematically summarized in **Figure 3**.

With deepening understanding of the mechanisms of integrins in pulmonary diseases, significant progress has been made in drug development targeting their distinct subtypes. Current therapeutic strategies primarily focus on monoclonal antibodies, small-molecule antagonists, peptide and cyclic peptide drugs, allosteric or silencing antagonists, as well as emerging drug conjugates and delivery technologies. Each class of drugs demonstrates potential in diseases such as pulmonary fibrosis, COPD, ARDS, asthma, and lung cancer, while also being extensively explored in other fields including ophthalmic diseases, osteoporosis, and solid tumors.

### 4.1 Monoclonal antibodies

Monoclonal antibodies represent the earliest and most specific class of drugs applied in integrin-targeted therapies. In pulmonary fibrosis, the α_v_β_6_ monoclonal antibody BG00011 (STX-100/3G9) has entered clinical trials, demonstrating the ability to slow lung function decline in patients with idiopathic pulmonary fibrosis (IPF). However, due to the occurrence of serious adverse events, clinical development of BG00011 in IPF has been discontinued (137). On the other hand, the α_4_β_1_ antibody natalizumab, initially approved for multiple sclerosis and inflammatory bowel disease, has been explored for asthma and chronic airway inflammation due to its mechanism of inhibiting immune cell infiltration (138). In oncology research, the α_v_β_3_ antibody medicinal drug MEDI-522 (Abergrin) was extensively evaluated for non-small cell lung cancer and other solid tumors but encountered development setbacks due to insufficient efficacy (139–141). Overall, monoclonal antibodies show significant promise in inflammatory diseases, particularly fibrosis, while still facing efficacy bottlenecks in oncology.

### 4.2 Small-molecule antagonists

Small-molecule antagonists represent another significant research direction due to their favorable pharmacokinetic properties and ease of oral or inhaled administration. Cilengitide, an RGD analog, was among the first large molecules to enter clinical trials. targeting α_v_β_3_/α_v_β_5_/α_5_β_1_. It is widely used in tumor research. Although it failed in Phase III trials for glioblastoma due to lack of efficacy (142, 143), it still demonstrated certain inhibitory effects on airway angiogenesis and inflammation in asthma models (144, 145). Risuteganib (Luminate), another α_v_ integrin inhibitor, has demonstrated efficacy in improving vascular leakage and fibrosis in clinical trials for age-related macular degeneration (AMD) and diabetic macular edema (DME) (146, 147). The α_v_β_6_ small-molecule inhibitor GSK3008348, administered via inhalation, reduces systemic drug exposure through cellular internalization and has demonstrated favorable outcomes in IPF models (148). The orally administered small molecule bexotegrast (PLN-74809) selectively inhibits both α_v_β_6_ and α_v_β_1_. Multiple Phase II studies in 2024–2025 demonstrated its favorable safety profile and suggestive trends of improvement in FVC decline and collagen-related imaging/molecular markers. α_v_β_6_-PET tracing confirmed the association between receptor occupancy and efficacy, providing evidence for precision stratification and companion diagnostics (149, 150). Additionally, broad-spectrum α_v_ inhibitors MK-0429 and GLPG0187 have undergone clinical evaluation in pulmonary fibrosis and solid tumors. Some trials demonstrated delayed disease progression, though efficacy requires further optimization (151, 152). The small-molecule α_v_β_1_ inhibitor CWHM-12 demonstrated the ability to reverse established fibrosis in a bleomycin-induced mouse model, suggesting its potential as a novel anti-fibrotic therapeutic (14, 153).

### 4.3 Cyclic peptides and peptide therapeutics

Cyclic peptides and peptide therapeutics have also been actively developed in recent years. The peptide small molecule JSM-6427 has been evaluated for ocular neovascular diseases such as AMD, demonstrating efficacy in preclinical stages (154). Another example is AXT-107, a synthetic 20-mer peptide derived from the non-collagenous domain of type IV collagen, which has now entered clinical trials for retinal vascular diseases (155). The macromolecular peptide TMV-7, which disassembles integrins by binding to α_IIb_β_3_, exerts antithrombotic effects with significantly lower bleeding risks than traditional anticoagulants. This suggests that similar strategies may be extended in the future to treat pulmonary vascular injury and thrombosis-related complications (156).

### 4.4 Allosteric antagonists and silent antagonists

The development of allosteric antagonists and silent antagonists stems from concerns about the side effects of traditional antagonists. Some RGD antagonists exhibit “reverse agonist” effects at low concentrations, paradoxically promoting angiogenesis in tumor models. To circumvent this issue, researchers designed small molecules that stably stabilize integrins in a low-affinity state, such as RUC-1, RUC-2, and RUC-4. RUC-4 has entered Phase II clinical trials in myocardial infarction patients, demonstrating advantages in reducing bleeding risk while maintaining antithrombotic efficacy (157, 158). Although these drugs are primarily applied in cardiovascular diseases, the same principles offer insights for optimizing α_v_ integrin-related pulmonary disease therapeutics.

### 4.5 Drug conjugates and novel delivery systems

Drug conjugates and novel delivery systems are also gaining prominence in integrin-targeting research. α_v_β_6_-targeted drug conjugates have been developed for lung cancer, leveraging the high expression of α_v_β_6_ in tumor tissues to achieve precise killing (159). Bionic nanovesicle delivery systems also show promising applications. For instance, combining β_2_ integrin-coated neutrophil membranes with anti-inflammatory drugs enables precise targeting of inflammatory pulmonary endothelium in ARDS, enhancing local efficacy while reducing systemic side effects (27, 81). Inhalation delivery represents another significant breakthrough. α_v_β_6_ inhibitors engineered as inhalable formulations can directly act on pulmonary lesions, effectively inhibiting associated airway fibrosis (112).

### 4.6 Combination therapy

Combination Therapy is another current research focus. Single-target therapies often struggle to address complex disease networks, making the combination of integrin inhibitors with immune checkpoint inhibitors particularly noteworthy in lung cancer treatment. By blocking the α_v_–TGF-β pathway, this approach significantly improves the immunosuppressive microenvironment and enhances the efficacy of PD-1/PD-L1 inhibitors (129, 160). In COPD and IPF, combining anti-inflammatory drugs with anti-fibrotic integrin inhibitors holds promise for dual benefits (3, 161). Concurrently, some studies propose strategies combining low-dose integrin agonists with chemotherapy—for instance, low-dose cilengitide promotes tumor angiogenesis, thereby enhancing gemcitabine delivery efficiency (162).

### 4.7 Therapeutic delivery routes and their impact on efficacy and safety

The route of administration is a critical determinant of both the therapeutic efficacy and safety profile of integrin-targeted agents. Inhaled delivery offers distinct advantages for lung-restricted diseases by achieving high local drug concentrations at the epithelial and interstitial interfaces while minimizing systemic exposure (163).This principle is exemplified by the inhaled α_v_β_6_ inhibitor GSK3008348, which was explicitly designed for direct pulmonary delivery and shows robust lung target engagement with markedly reduced systemic bioavailability (164); PET imaging further confirms dose-dependent α_v_β_6_ receptor occupancy in IPF patients following nebulized administration (165). In contrast, orally administered inhibitors, such as the dual α_v_β_6_/α_v_β_1_ antagonist bexotegrast, provide systemic exposure that supports whole-lung distribution but also necessitates careful dose optimization to avoid off-target blockade of integrins in non-pulmonary tissues (150). Systemic intravenous administration illustrates the clearest safety trade-offs. The α_4_-integrin monoclonal antibody natalizumab, for example, effectively suppresses leukocyte trafficking but simultaneously impairs CNS immune surveillance, leading to an increased risk of progressive multifocal leukoencephalopathy (PML) during long-term treatment (166, 167). Similarly, systemic inhibition of platelet integrin α_IIb_β_3_ with glycoprotein IIb/IIIa antagonists—including both intravenous and oral formulations—has been repeatedly associated with acute immune-mediated thrombocytopenia and major bleeding due to widespread integrin blockade on circulating platelets (168). These clinical experiences collectively highlight that local delivery routes, such as inhalation, can greatly enhance on-target efficacy while reducing systemic toxicities, whereas systemic routes require vigilant monitoring for unintended integrin inhibition across diverse vascular and immune compartments.

Overall, anti-integrin therapies have evolved from early single antagonists to diverse strategies encompassing monoclonal antibodies, small molecules, peptides, allosteric antagonists, and novel drug delivery approaches. These diverse agents demonstrate therapeutic potential across pulmonary fibrosis, ARDS, COPD, asthma, and lung cancer, with some expanding into ophthalmology and cardiovascular medicine. Moving forward, achieving disease-specific, organ-targeted, and personalized drug delivery will be pivotal to advancing the true translational application of integrin inhibitors.

## 5 Outlook and challenges

Although therapeutic strategies targeting integrins show tremendous potential in pulmonary diseases, their clinical application still faces numerous challenges. First, integrins perform vital functions in normal physiological processes, including maintaining tissue homeostasis, promoting wound healing, and regulating immune surveillance. Excessive or non-specific inhibition may lead to severe side effects (169). For instance, anti-α_L_β_2_ monoclonal antibodies previously used for immune-related diseases were discontinued due to inducing progressive multifocal leukoencephalopathy (PML), highlighting immunosuppression-related risks in integrin-targeted therapies (170). Second, the large integrin family exhibits functional redundancy, with different subtypes potentially playing compensatory roles in inflammatory or fibrotic processes. Loike et al. demonstrated that when β_2_ integrins (LFA-1/Mac-1) were blocked, neutrophils retained the ability to migrate through fibrin gels via β_1_ integrins, particularly α_5_;β_1_, indicating that β_5_-dependent matrix interactions can sustain interstitial locomotion even when β_2_-mediated adhesion is impaired (171).This implies that single-target approaches often fail to achieve complete efficacy, while multi-target combination blockade may exacerbate systemic side effects. Precisely identifying key integrin subtypes and their spatiotemporal actions at different disease stages represents a core scientific challenge for achieving effective interventions.

Furthermore, the lack of standardized efficacy evaluation criteria significantly constrains clinical advancement. Current pulmonary fibrosis trials predominantly rely on pulmonary function and imaging metrics, which often lack sensitivity and specificity. Future efforts should integrate high-resolution CT quantitative analysis, serum biomarkers (e.g., PRO-C3, periostin), and emerging molecular imaging techniques into comprehensive evaluation systems to capture drug effects earlier and more accurately. In drug development, small-molecule antagonists require further optimization in stability, selectivity, and pulmonary delivery efficiency, while large-molecule therapies like monoclonal antibodies need to reduce immunogenicity and explore the feasibility of local administration.

Overall, integrin-targeted therapies are at a stage where opportunities and challenges coexist. Future progress requires close integration of basic and clinical research: on one hand, deepening the understanding of the precise mechanisms of integrin action across different diseases and disease stages to provide clearer target rationale for drug design; on the other hand, leveraging advances in pharmaceutics and delivery technologies to develop safe, effective, and personalized intervention strategies. As these challenges are progressively addressed, integrin-targeting therapies hold promise as a novel breakthrough for treating multiple pulmonary diseases, including ARDS, IPF, COPD, asthma, and lung cancer.
